# Factors Affecting Obesity and Waist Circumference Among US Adults

**DOI:** 10.5888/pcd16.180220

**Published:** 2019-01-03

**Authors:** Daniel Kim, Wei Hou, Fusheng Wang, Chrisa Arcan

**Affiliations:** 1 Department of Biomedical Informatics and Department of Computer Science, Stony Brook University, Stony Brook, New York; 2 Department of Family, Population, and Preventive Medicine, Stony Brook University, Stony Brook, New York

## Abstract

**Introduction:**

Physical activity, sedentary activity, and food intake affect waist circumference and obesity among adults; however, the relationship is unclear. The objective of our study was to explore how these factors affect waist circumference and obesity prevalence among adults.

**Methods:**

We used cross-sectional data from the National Health and Nutrition Examination Survey 2013–2014 on 4,118 adults, 49% men and 51% women, aged 20 to 64 (mean age, 42). Weighted logistic regression models were fitted for abdominal obesity or obesity status and adjusted for variables of demographic characteristics, food intake, types of physical and sedentary activity, television and video viewing, and computer use. Analyses were stratified by sex.

**Results:**

Of the 4,118 people studied, 39% were obese (body mass index ≥30) and 55% had a high-risk waist circumference (hereinafter, abdominal obesity: men, ≥120 cm; women, ≥88 cm). People who watched television or videos 2 hours or more per day had increased odds of being abdominally obese (men, odds ratio [OR], 1.96; 95% confidence interval [CI], 1.29%–2.98%; women, OR, 1.66; 95% CI, 1.06%–2.59%) or obese (men, OR, 2.17; 95% CI, 1.18%–4.02%; women, OR, 1.66; 95% CI, 1.12%–2.48%). After adjusting for types of physical activity, associations remained significant only among men. Moderate recreational physical activity for 150 minutes or more a week versus 149 minutes or less was associated with reduced odds of abdominal obesity for both men (OR, 0.44; 95% CI. 0.22%–0.89%) and women (OR, 0.98; 95% CI, 0.23%–0.67%). Consuming meals prepared away from home was associated with high odds of obesity among women (OR, 1.67; 95% CI, 1.08%–2.58%).

**Conclusion:**

Watching television and videos was positively associated with prevalence of abdominal obesity and obesity among men and women. Prevalence remained significant only among men with inclusion of physical activity. Further study is needed of the differences between the sexes in how physical and sedentary activity and food consumption are associated with obesity.

## Introduction

Poor diet, low levels of physical activity, and high levels of sedentary activities are risk factors for obesity. Because diet and activity are modifiable factors, addressing this risk requires an understanding of their contribution to obesity. A meta-study of National Health and Nutrition Examination Survey (NHANES) data sets showed leisure-time physical activity to be inversely associated with obesity (1). In the Coronary Artery Risk Development in Young Adults study, transportation-related physical activity was shown to lessen or reverse effects of weight gain (2). Multiple studies have shown an association between sedentary activity and increased rates of obesity, independent of physical activity (3,4,5).

Abdominal obesity (waist circumference ≥102 cm for men and ≥88 cm for women), independent of body mass index (BMI) (calculated as weight in kilograms divided by height in meters squared), has been associated with major chronic diseases and all-cause mortality (6). Abdominal fat, rather than total body fat, was found to be the cause of the systemic inflammation that contributes to chronic disease (7). Intervention and population studies have indicated that being sedentary or having a low fitness level is also associated with visceral fat accumulation (7,8,9). Various sedentary activities are differentially associated with cardiometabolic factors, including abdominal obesity (10). Associations between sedentary activity and obesity differ between the sexes. A large population study of employed Canadian adults found occupation-related sedentary activity to be associated with BMI and waist circumference among men irrespective of leisure-time physical activity (11).

Little research has been conducted on specific types of physical activity and their relationship to obesity, and few studies have examined how waist circumference (hereinafter, abdominal obesity) is related to physical activity, sedentary activity, and diet. To address this information gap, we examined how adult obesity and abdominal obesity is associated with physical activity, sedentary activity, and consumption of meals prepared outside the home (ie, from conventional or fast-food restaurants, food stands or trucks, grocery stores, or vending machines). We hypothesized that all types of physical activity have an inverse relationship with obesity and abdominal obesity, and frequent sedentary activity and consumption of meals prepared outside the home increase the risk of both conditions. Such information can guide public health policies and interventions.

## Methods

We examined associations between obesity and abdominal obesity and types of physical activity, sedentary activity, and diet among US adults aged 20 to 64 who participated in the National Health and Nutrition Examination Survey (NHANES) 2013–2014. NHANES collects survey-based data annually to assess variables related to health and nutrition among the noninstitutionalized, civilian population of the United States (12).

Demographic variables were age, race/ethnicity, education, employment (employed, unemployed), and marital status (married, unmarried); all variables were self-reported. To ensure representation of minority groups, NHANES oversamples certain populations, such as Hispanic, black, and Asian populations; low-income populations; and the elderly (13). NHANES uses the following stages in sample selection: 1) counties or small groups of counties (primary sampling units), 2) segments within sampling units, 3) households within segments, and 4) individuals within households (12). Of the 10,175 individuals in the NHANES 2013–2014 data set, we excluded children aged 0 to 19 years, adults 65 years of age or older, pregnant women, underweight adults (BMI <18.5), morbidly obese adults (BMI >60), and participants with missing, “don’t know,” or null responses, for a sample size of 4,118 for our analysis. The Institutional Review Board for the Ethics Review Board of the National Center for Health Statistics approved NHANES data collection and allowed data files to be posted on their website for public use (14). Written informed consent was obtained from participants before collection.

### Dependent variables

We examined prevalence of obesity and abdominal obesity as outcome variables in independent analyses. Height and weight were collected in a mobile examination center by using standardized protocols. From those measurements, we calculated BMI and rounded it to the nearest tenth. Obesity was defined as BMI at or above 30. Waist circumference was measured with a tape measure at the uppermost lateral border of the hip crest (15). Waist circumferences of 120 cm or more for men and 88 cm or more for women were considered high risk and termed abdominal obesity.

### Independent variables

Measurements of physical activity arising from work, recreation, and transportation were used to assess the effect of each on total obesity and abdominal obesity. Physical activity was based on the Centers for Disease Control and Prevention’s (CDC’s) physical activity guidelines for adults (16). According to CDC’s guidelines, to achieve substantial health benefits, adults should engage in at least 150 minutes a week of moderate physical activity or 75 minutes a week of vigorous physical activity. Vigorous activity was defined as activity that caused large heart rate or breathing increases, and moderate activity was defined as activity that caused small increases. Thus, we dichotomized work-related and recreational physical activity variables to vigorous (<75 vs ≥75 min/wk) or moderate (<150 vs ≥150 min/wk). Transportation was defined as walking or bicycling to get to and from places. Transportation-related physical activity was dichotomized to less than 75 minutes per week versus 75 minutes or more per week.

Overall sedentary activity was assessed by asking participants to enter the total minutes each day they spent sitting in various settings: school, at home, getting to and from places, or with friends, including time spent sitting at a desk, traveling in a car or bus, reading, playing cards, watching television, or using a computer. Responses ranged from 0 to 1,200 minutes per day. Three categories of daily sedentary activity were created (0 to 360 minutes, 360 to 540 minutes, or 540 minutes or more) on the basis of categories used in a previous study of leisure time among US adults (17) and median statistics on time spent in sedentary activity in the United States (18).

Television viewing (including watching videos) and computer use were separately examined as sedentary activities. Participants were asked to report the average hours per day in the past 30 days they spent sitting and watching television or using a computer; 6 response categories ranged from less than 1 hour to 5 hours or more. Responses for television watching or computer use variables were dichotomized to less than 2 hours a day versus 2 hours or more per day, because less than 2 hours per day of television watching is associated with gains in life expectancy (19).

Two variables related to types of meals consumed in the past 30 days were included in the model: food prepared outside the home and frozen meals or pizza consumed in the home. Participants were asked to report the number of meals they consumed in the past 7 days that were prepared outside the home. Responses ranged from none to more than 21. Participants were also asked to report how often they ate frozen meals or pizza at home during the past 30 days. Responses ranged from never to 180 times. Responses were dichotomized to 0 to 2 versus 3 or more times per week on the basis of the Eat Among Teens survey, which measured fast food’s influence on families when consumed 3 times per week or more (20).

### Statistical analysis

Statistical analyses were performed by using sample weights and stratum as designed and collected by the National Center for Health Statistics for complex sampling to provide nationally representative estimates and to address oversampling, nonresponse, and noncoverage. We used weighted analysis of variance for continuous variables and χ^2^ test for categorical variables to perform univariate analysis to evaluate independent associations between population characteristics and obesity, abdominal obesity, and sex. Weighted logistic regression models were fitted for obesity status (obese, yes/no) or abdominal obesity risk status (high or low) as the dependent variables. Models were developed for each type of physical activity, because small sample sizes precluded simultaneous inclusion of all physical activity variables. For logistic regression, physical activity, sedentary activity, and television watching or computer use variables were transformed into categorical variables according to CDC research guidelines or our defined high-risk and low-risk groups. All logistic regression analyses were stratified by sex.

Six models were created for each outcome (obesity and abdominal obesity). The base model included all the demographic variables, 2 food intake variables, general sedentary activity, and television and computer use variables. Each of the other models included the base model adjusted for each type of physical activity (ie, moderate, vigorous, transportation) as an independent variable. These models were constructed by adding the additional independent variable to our base model. Odds ratios (ORs) and their 95% confidence intervals (CIs) were estimated and tested for significance on the basis of logistic regression. Two-way interactions between physical activities and other characteristics (eg, interaction between physical activity and obesity status for abdominal obesity model) were evaluated in the weighted logistic regressions; however, because of sparsely distributed physical activity data, no valid model-fitting could be achieved with the inclusion of the interactions (ie, convergence or maximum likelihood estimates could not be obtained). Therefore, all interactions were excluded from final models. Calculations and model creations were performed by using SAS version 9.4 (SAS Institute, Inc).

## Results

Of the 4,118 participants included in the study, 69% were white, 55% were married, and 51% were women; the mean age of participants was 42 (Table 1). More women (42%) than men (35%) were obese, and more women (66%) than men (44%) had abdominal obesity. More men engaged in transportation physical activity than women (57% men vs 47% women). A higher percentage of men (70%) than women (65%) watched television more than 2 hours a day. Also, more men (60%) than women (45%) consumed meals prepared outside the home 3 times or more a week.

**Table 1:** Sociodemographic and Physical Characteristics by Factors Affecting Obesity and Waist Circumference (Abdominal Obesity) Among US Adults Aged 20 to 64 (N = 4,118), NHANES 2013–2014^a^

Characteristic	Total	Men	Women	*P* Value^b^
**Respondents, no. (%)**	4,118 (100)	2,014 (49)	2,104 (51)	NA
**Age, mean, y**	42	42	42	.12
**Race/ethnicity**				
White	1,634 (69)	810 (69)	824 (69)	.76
Black	859 (13)	421 (12)	438 (14)	.007
Hispanic	988 (18)	474 (19)	514 (18)	.35
**Education**				
Less than high school diploma	814 (14)	432 (15)	382 (13)	.11
High school graduate	2,244 (55)	1,061 (53)	1,183 (57)	
Some college	1,059 (31)	521 (31)	538 (30)	
**Married**	2,125 (55)	1,081 (57)	1,044 (53)	.001
**Employed**	2,825 (72)	1,517 (80)	1,308 (65)	<.001
**Physical characteristics**				
Overweight or obese	2,894 (71)	1,447 (75)	1,447 (66)	<.001
Obese (body mass index^c^ ≥30)	1,599 (39)	686 (35)	913 (42)	.004
Abdominally obese^d^	2,159 (55)	798 (44)	1,361 (66)	<.001
**Work-related physical activity, min/wk**				
Vigorous (75–149)	758 (88)	533 (90)	225 (84)	.05
Moderate (150–299)	1,146 (79)	632 (80)	514 (78)	.57
**Recreational physical activity, min/wk**				
Vigorous (75–149)	927 (86)	552 (86)	375 (86)	.92
Moderate (150–299)	808 (46)	404 (47)	404 (46)	.68
**Transportation physical activity ≥75 min/wk**	601 (53)	349 (57)	252 (47)	.02
**Total sedentary activity, min/d**				
0–359	1,504 (35)	724 (34)	780 (36)	.44
360–539	1,474 (35)	736 (36)	738 (34)	
≥540	1,135 (30)	552 (30)	583 (30)	
**Television viewing ≥2 hr/d**	2,763 (67)	1,391 (70)	1,372 (65)	.007
**Computer use ≥2 hr/d**	2,273 (51)	1,155 (53)	1,118 (50)	.11
**Diet**				
Ate meals prepared away from home ≥3 times/wk	1,973 (52)	1,111 (60)	862 (45)	<.001
Ate frozen meals or pizza in past 30 days ≥3 times/wk	870 (24)	413 (23)	457 (25)	.23
**Smoker**	973 (53)	527 (49)	446 (57)	.009

a Values are number (percentage) unless otherwise indicated. Sample size variations are due to incidental missing values in returned surveys; thus, not all values sum to total respondents.

b Weighted and stratified χ^2 ^tests were used to compare sexes and to generate *P* values.

c Calculated as weight in kilograms divided by height in meters squared.

d Waist circumference ≥120 cm for men and ≥88 cm for women.

In the base model, adults who watched television 2 hours or more per day had higher odds of abdominal obesity (men, OR, 1.96; 95% CI, 1.29–2.98; women, OR, 1.66; 95% CI, 1.06–2.59) and obesity (men, OR, 2.17; 95% CI, 1.18–4.02; women, OR, 1.66; 95% CI, 1.12–2.48) than those who watched 2 hours or less (Table 2). In the model that adjusted for moderate work-related physical activity, only men who watched television more than 2 hours a day had higher odds of abdominal obesity (OR, 2.68; 95% CI, 1.30–5.53) than men who watched less than 2 hours daily. In the model that adjusted for transportation physical activity, only men who watched television 2 hours or more per day had higher odds of abdominal obesity (OR, 3.24; 95% CI, 1.28–8.20) or obesity (OR, 3.28; 95% CI, 1.20–8.96) than men who watched less than 2 hours (Table 3). In the model that adjusted for vigorous recreational physical activity, watching television 2 hours or more per day was also associated with higher odds (OR, 3.87; 95% CI, 1.53–9.78) of obesity among men only. In the model that adjusted for transportation activity, men who engaged in sedentary activity for 540 minutes or more per day had higher odds of abdominal obesity after adjusting for transportation physical activity (OR, 2.84; 95% CI, 0.93–8.64) than men who engaged in sedentary activities 359 minutes or less per day.

**Table 2:** Risk of Abdominal Obesity and Obesity^a^ by Behavior Among Adults Aged 20 to 64 (N = 4,118) Who Engaged in Work-Related Physical Activity, National Health and Nutrition Examination Survey, 2013–2014^b^

Behavior	Base Model^c^ (n = 1,287)		Base Model^c^ With Vigorous Work-Related Physical Activity^d^ (n = 363)		Base Model^c^ With Moderate Work-Related Physical Activity^e^ (n = 548)	
	Men	Women	Men	Women	Men	Women
**Abdominal Obesity**						
Ate meals prepared away from home <3 vs ≥3 times/wk	1.35 (0.88–2.07)	1.58 (0.87–2.85)	1.35 (0.62–2.95)	1.36 (0.68–2.71)	1.06 (0.65–1.75)	1.72 (0.73–4.04)
Ate frozen meals/pizza in past 30 days <3 vs ≥3 times/wk	1.15 (0.71–1.87)	1.27 (0.76–2.11)	1.28 (0.59–2.74)	0.86 (0.21–3.49)	1.27 (0.60–2.70)	1.20 (0.51–2.84)
Sedentary activity ≤359 vs 360–539 min/d	1.24 (0.71–2.19)	0.95 (0.57–1.59)	1.97 (0.90–4.33)	1.40 (0.17–11.52)	1.46 (0.65–3.28)	1.26 (0.53–3.01)
Sedentary activity ≤359 vs ≥540 min/d	1.38 (0.81–2.33)	0.85 (0.38–1.89)	2.35 (0.79–6.99)	0.54 (0.07–4.03)	1.01 (0.45–2.26)	0.65 (0.21–2.02)
Watching television or videos <2 vs ≥2 hr/d	1.96 (1.29–2.98)^f^	1.66 (1.06–2.59)^f^	1.58 (0.77–3.26)	0.96 (0.34–2.73)	2.68 (1.30–5.53)^f^	1.32 (0.71–2.44)
Computer/video game usage <2 vs ≥2 hr/d	1.11 (0.67–1.85)	1.27(0.85–1.89)	0.79 (0.34–1.85)	1.90 (0.83–4.39)	0.70 (0.29–1.69)	1.19 (0.63–2.24)
Vigorous work-related physical activity ≤74 vs ≥75 min/wk	—	—	0.25 (0.08–0.77)^f^	1.26 (0.50–3.19)	—	—
Moderate work-related physical activity ≤149 vs ≥150 min/wk	—	—	—	—	1.31 (0.64– 2.67)	0.86 (0.30– 2.42)
**Obesity**						
Ate meals prepared away from home <3 vs ≥3 times/wk	1.07 (0.69–1.68)	1.67 (1.08–2.58)^f^	0.89 (0.47–1.69)	1.02 (0.44–2.38)	1.03 (0.61–1.73)	2.37 (1.09–5.13)^f^
Ate frozen meals/pizza in past 30 days <3 vs ≥3 times/wk	1.34 (0.90–2.00)	0.96 (0.55–1.70)	1.45 (0.58–3.59)	1.38 (0.56–3.42)	1.33 (0.76–2.35)	1.48 (0.59–3.70)
Sedentary activity ≤359 vs 360–539 min/d	1.47 (0.82–2.63)	1.41 (1.03–1.93)^f^	1.57 (0.75–3.26)	0.60 (0.16–2.29)	2.19 (0.83–5.74)	1.26 (0.60–2.66)
Sedentary activity ≤359 vs ≥540 min/d)	1.22 (0.63–2.36)	1.14 (0.60–2.16)	1.56 (0.54–4.47)	0.63 (0.11–3.61)	0.97 (0.40–2.34)	0.52 (0.17–1.64)
Watching television or videos <2 vs ≥2 hr/d	2.17 (1.18–4.02)^f^	1.66 (1.12–2.48)^f^	1.83 (0.94–3.56)	1.20 (0.35–4.11)	2.37 (1.09–5.14)^f^	1.28 (0.81–2.02)
Computer or video game use <2 vs ≥2 hr/d	1.15 (0.75–1.75)	1.15 (0.72–1.83)	0.64 (0.27–1.50)	1.44 (0.50–4.13)	0.60 (0.25–1.45)	1.27 (0.71–2.26)
Vigorous work-related physical activity ≤74 vs ≥75 min/wk	—		1.57 (0.65–3.82)	1.11 (0.24–5.05)	—	—
Moderate work-related physical activity ≤149 vs ≥150 min/wk	—	—	—	—	2.46 (1.55–3.90)^f^	0.70 (0.34–1.44)

Abbreviation: —, not applicable.

a Abdominal obesity was defined as a waist circumference ≥120 cm for men and ≥88 cm for women. Obesity was defined as a body mass index (calculated as weight in kilograms divided by height in meters squared) of ≥30.

b Values are odds ratio (95% confidence interval). Odds ratios were calculated by using logistic regression.

c Includes all demographic variables (age, race/ethnicity, education, employment, and marital status), 2 food intake variables (consumption of meals prepared away from home and consumption of frozen meals or pizza at home), and variables for general sedentary activity, television and video viewing, and computer use.

d Activity that causes large increases in heart rate or breathing.

e Activity that causes small increases in heart rate or breathing.

f Significant at *P* < .05.

**Table 3:** Risk of Abdominal Obesity and Obesity^a^ by Behavior Among Adults Aged 20 to 64 (N = 4,118) Who Engaged in Recreational and Transportation Physical Activity, National Health and Nutrition Examination Survey, 2013–2014^b^

Behavior	Base Model^c^ With Vigorous Recreational Physical Activity^d^ (n = 266)		Base Model^c^ With Moderate Recreational Physical Activity^e^ (n = 498)		Base Model^c^ With Transportation Physical Activity^f^ (n = 335)	
	Men	Women	Men	Women	Men	Women
**Abdominal Obesity**						
Ate meals prepared away from home <3 vs ≥3 times/wk	0.98 (0.43–2.22)	1.27 (0.21–7.46)	1.13 (0.72–2.40)	1.55 (0.55–4.39)	1.43 (0.60–3.44)	1.85 (0.34–10.04)
Ate frozen meals/pizza in past 30 days <3 vs ≥3 times/wk	1.21 (0.37–3.96)	3.56 (2.18–5.81)^g^	0.47 (0.19–1.18)	1.81 (0.55–3.87)	1.26 (0.37–4.30)	1.31 (0.34–5.08)
Sedentary activity ≤359 vs 360–539 min/d	1.64 (0.93–2.92)	0.86 (0.21–3.50)	1.24 (0.72–2.11)	0.52 (0.15–1.85)	1.24 (0.45–3.46)	0.57 (0.13–2.47)
Sedentary activity ≤359 vs ≥540 min/d	0.59 (0.11–3.26)	1.73 (0.61–4.95)	2.05 (0.72–5.81)	0.26 (0.07–1.01)	2.84 (0.93–8.64)^g^	0.13 (0.03–0.54)^g^
Watching television or videos <2 vs ≥2 hr/d	3.79 (0.85–16.85)	0.48 (0.10–2.42)	1.95 (0.94–4.05)	1.17 (0.54–2.52)	3.24 (1.28–8.20)^g^	1.40 (0.49–4.06)
Computer or video game use <2 vs ≥2 hr/d	1.36 (0.45–4.12)	1.83 (0.50–6.75)	0.93 (0.40–2.18)	1.26 (0.61–2.59)	0.47 (0.19–1.18)	2.29 (0.61–8.65)
Vigorous recreational physical activity ≤74 vs ≥75 min/wk	0.31 (0.11–0.88)^g^	0.60 (0.13–2.81)	—	—	—	—
Moderate recreational physical activity ≤149 vs ≥150 min/wk	—	—	0.44 (0.22–0.89)^g^	0.38 (0.23–0.67)^g^	—	—
Transportation physical activity ≤149 vs ≥150 min/wk	—	—	—	—	0.58 (0.23–1.46)	0.71 (0.54–3.44)
**Obesity**						
Ate meals prepared away from home <3 vs ≥3 times/wk	0.83 (0.54–1.28)	3.22 (0.72–14.41)	1.28 (0.65–2.50)	1.61 (0.75–3.44)	1.64 (0.69–3.87)	1.75 (0.79–3.89)
Ate frozen meals/pizza in past 30 days <3 vs ≥3 times/wk	1.16 (0.54–2.49)	2.27 (0.72–7.11)	0.87 (0.42–1.83)	1.14 (0.71–1.82)	1.79 (0.62–5.11)	1.69 (0.46–6.17)
Sedentary ≤359 vs 360–539 min/d	2.10 (0.82–5.36)	1.67 (0.25–11.30)	1.52 (0.90–2.56)	1.59 (0.93–2.73)	1.25 (0.51–3.07)	0.88 (0.28–2.74)
Sedentary ≤359 vs ≥540 min/d	0.85 (0.14–5.26)	1.29 (0.17–9.79)	1.80 (0.46–7.02)	0.60 (0.24–1.51)	3.06 (0.97–9.58)	0.40 (0.13–1.25)
Watching television or videos <2 vs ≥2 hr/d	3.87 (1.53–9.78)^g^	0.81 (0.14–4.61)	1.98 (0.84–4.69)	1.11 (0.46–2.70)	3.28 (1.20–8.96)^g^	1.23 (0.51–2.99)
Computer or video game use <2 vs ≥2 hr/d	0.89 (0.33–2.40)	0.70 (0.05–9.53)	1.01 (0.48–2.14)	0.83 (0.38–1.81)	0.59 (0.25–1.42)	1.94 (0.82–4.57)
Vigorous recreational physical activity ≤74 vs ≥75 min/week	0.39 (0.14–1.08)	0.85 (0.15–4.74)	—	—	—	—
Moderate recreational physical activity ≤149 vs ≥150 min/week	—	—	0.97 (0.40–1.86)	0.48 (0.27–0.84)^g^	—	—
Transportation physical activity ≤149 vs ≥150 min/wk	—	—	—	—	0.73 (0.32–1.67)	0.59 (0.22–1.59)

Abbreviation: —, not applicable.

a Abdominal obesity was defined as a waist circumference ≥120 cm for men and ≥88 cm for women. Obesity was defined as a body mass index (calculated as weight in kilograms divided by height in meters squared) of ≥30.

b Values are odds ratio (95% confidence interval). Odds ratios were calculated by using logistic regression.

c Includes all demographic variables (age, race/ethnicity, education, employment, and marital status), 2 food intake variables (consumption of meals prepared away from home and consumption of frozen meals or pizza at home), and variables for general sedentary activity, television and video viewing, and computer use.

d Activity that causes large increases in heart rate or breathing.

e Activity that causes small increases in heart rate or breathing.

f Walking or bicycling for getting to and from places.

g Significant at *P* < .05.

Engaging in moderate recreational physical activity for 150 minutes or more per week versus 149 minutes or less was associated with reduced odds of abdominal obesity for both men (OR, 0.44; 95% CI, 0.22–0.89) and women (OR, 0.38; 95% CI, 0.23–0.67) (Table 3) and with lower odds of obesity among women only (OR, 0.48; 95% CI, 0.27–0.84). Engaging in vigorous work-related or vigorous recreational activity was protective against abdominal obesity for men only (work-related, OR, 0.25; 95% CI, 0.08–0.77 [Table 2]; recreational, OR, 0.31; 95% CI, 0.11–0.88 [Table 3]). In the model that adjusted for transportation-related physical activity, an inverse association between overall sedentary activity and abdominal obesity was found among women only (OR, 0.13, 95% CI, 0.3–0.54).

Among women, eating meals prepared away from home 3 days a week or more versus less than 3 days was associated with higher odds of obesity (OR, 1.67; 95% CI, 1.08–2.58) in the base model and after adjusting for moderate work-related physical activity (OR, 2.37; 95% CI, 1.09–5.13) (Table 2). Eating frozen meals or pizza 3 or more times a week versus less than 3 days was associated with increased odds of abdominal obesity among women (OR, 3.56; 95% CI, 2.18–5.81) (Table 3) after adjustment for vigorous recreational physical activity. We found no association among men between eating meals prepared away from home and obesity or abdominal obesity.

## Discussion

Although many types of physical activity were associated with reduced risk of obesity and abdominal obesity as our hypothesis predicted, work-based physical activity was not. Sedentary activity in general was not linked to increased risk, in opposition to our hypothesis; only excess television watching was linked to the risk of obesity and abdominal obesity. Unhealthy meals did not increase obesity risk, in complete contrast to what we initially hypothesized. When considering public health implications, our models show that public health initiatives must focus on increasing recreational physical activity and decreasing television-based sedentary activity.

Other studies measured associations between types of physical activity and obesity, but connections between specific types of activity in relationship to work and recreational physical activity have rarely been studied. One study used accelerometer data to evaluate NHANES 2003–2006 and found strong associations between moderate and vigorous physical activity and obesity (21), although our study did not. This accelerometer-based study used a different method and a different time period than we did, although these differences may not be strong enough to account for the difference in results.

The consistent link between excess television viewing and risk for abdominal obesity and obesity among both sexes and the persistent link among men after incorporating types of physical activity indicates that efforts to prevent or reduce weight gain should focus on reducing television watching independent of increasing physical activity. Our findings agree with other studies showing the consistent associations between obesity risk and sedentary activity, physical activity, and waist circumference. A longitudinal study found a synergistic effect of reduced moderate-to-vigorous physical activity and increased television viewing on increases in waist circumference among a large sample of adults (22). In the Nurse’s Health Study, women with high levels of sedentary activity, especially television watching, had a significantly elevated risk of obesity, independent of physical activity levels; even small increases in moderate activity substantially lowered their obesity risk (23).

Our findings showed strengthening of the associations between television watching and measures of obesity (waist circumference and BMI) after adjusting for various types of physical activity, especially for men. A large longitudinal Canadian study found a strong association among men between occupational sedentary behaviors and obesity indicators after adjusting for vigorous physical activity (11). Little research exists about computer use among adults because most studies focus on youth and adolescents. Our study agrees somewhat with a study of 2,650 adults in Adelaide, Australia, that demonstrated that participants with high internet and other computer use were much more likely to be obese than those without. That study did not examine the effects of television watching, although it considered leisure-time sedentary activity (24).

Our finding of a persistent association among men between sedentary activity and measures of obesity after adjusting for various types of physical activity warrants further investigation. A systematic review suggested that snacking or other dietary intake during television viewing may mediate this association (25). Because the prevalence of excess television viewing was similar among men and women in our study, related behaviors (eg, consumption of alcohol or nutrient-dense snacks) need to be explored to more accurately establish the differences between the sexes in the association between television viewing and obesity and abdominal obesity (11). Furthermore, obesity prevention programs should explore creative ways to replace some television viewing time for men with other less sedentary activities. One study showed that replacing time spent in sedentary behavior with the same time in light or moderate-to-vigorous physical activity resulted in a decrease in waist circumference and cardiovascular biomarkers (26).

A surprising finding in our study was the inverse association among women between engaging in overall sedentary activities and abdominal obesity after adjusting for transportation physical activity. A similar result was found in a study by Nicholas and colleagues, indicating an inverse association between sedentary time and waist-to-hip ratio (11).

In our study, consuming meals prepared away from home was linked to increased abdominal obesity and obesity among women, irrespective of types of physical activity. This finding indicates that dietary intake may differentially influence weight gain for women compared with men. Furthermore, the separately significant associations between higher consumption of meals prepared outside the home, overall sedentary activity, and higher frequency of television watching (in our base model) with obesity prevalence substantially increases women’s risk for obesity-related chronic diseases. However, our models that adjusted for various types of physical activity attenuated these associations, indicating benefits of even moderate physical activity for women. A meta-analysis that focused on long-term walking patterns in adults concluded that walking can prevent or reduce common weight gains (2).

A strength of our study is its large sample size, which provided the statistical power to explore sex-specific associations. Using the NHANES data set — which represents a cross-section of the entire US population, including ethnic and underserved populations — enhances the generalizability of our findings. Another strength of our study is the inclusion of different types of physical activity, which we explored along with sedentary activity, dietary practices, and sociodemographic characteristics.

A limitation of our study is its cross-sectional design, which precludes a causal inference of our findings. Our study also suggests that the associations of physical activity, sedentary activity, and obesity outcomes might be bidirectional. As previously indicated, testing for interactions between physical activity and other variables was not possible. Social desirability bias may have lead respondents to underestimate their sedentary activity or overestimate their physical activity, causing further inaccuracies. Such inaccuracies are unavoidable in a study based on the NHANES data set.

Our findings suggest that television watching is positively associated with prevalence of abdominal obesity and obesity among both men and women. These associations persisted even after adjusting for various types and levels of physical activity, especially among men, suggesting the concurrence of other obesogenic behaviors, such as snacking or alcohol consumption while watching television. Future studies should explore these potential confounders. Our findings also show that consuming meals prepared away from home or frozen prepared meals was associated with risk of abdominal obesity and obesity among women only. This finding indicates that dietary intake may differentially influence weight gain in women compared with men. Although most of our findings are in agreement with other studies, little research exists that explores differences between the sexes in the associations between various types of sedentary and physical activity behaviors and obesity measures, adjusting for underlying factors such as food intake, that are linked to these activity behaviors.
